# Prescribing Hemodialysis or Hemodiafiltration: When One Size Does Not Fit All the Proposal of a Personalized Approach Based on Comorbidity and Nutritional Status

**DOI:** 10.3390/jcm7100331

**Published:** 2018-10-08

**Authors:** Giorgina Barbara Piccoli, Louise Nielsen, Lurilyn Gendrot, Antioco Fois, Emanuela Cataldo, Gianfranca Cabiddu

**Affiliations:** 1 Néphrologie Centre Hospitalier Le Mans, Avenue Roubillard 196, 72000 Le Mans, France; lnielsen@ch-lemans.fr (L.N.); lgendrot@ch-lemans.fr (L.G.); afois@ch-lemans.fr (A.F.); emanuela.cataldo@gmail.com (E.C.); 2 Dipartimento di Scienze Cliniche e Biologiche, University of Torino, Ospedale san Luigi, Regione Gonzole, 10100 Torino, Italy; 3 Nefrologia, Università Aldo Moro, Piazza Umberto I, 70121 Bari, Italy; 4 Nefrologia Ospedale Brotzu, Piazzale Alessandro Ricchi, 1, 09134 Cagliari, Italy; gianfranca.cabiddu@tin.it

**Keywords:** hemodialysis, hemodiafiltration, life expectancy, incremental dialysis, daily hemodialysis, personalization of treatment

## Abstract

There is no simple way to prescribe hemodialysis. Changes in the dialysis population, improvements in dialysis techniques, and different attitudes towards the initiation of dialysis have influenced treatment goals and, consequently, dialysis prescription. However, in clinical practice prescription of dialysis still often follows a “one size fits all” rule, and there is no agreed distinction between treatment goals for the younger, lower-risk population, and for older, high comorbidity patients. In the younger dialysis population, efficiency is our main goal, as assessed by the demonstrated close relationship between depuration (tested by kinetic adequacy) and survival. In the ageing dialysis population, tolerance is probably a better objective: “good dialysis” should allow the patient to attain a stable metabolic balance with minimal dialysis-related morbidity. We would like therefore to open the discussion on a personalized approach to dialysis prescription, focused on efficiency in younger patients and on tolerance in older ones, based on life expectancy, comorbidity, residual kidney function, and nutritional status, with particular attention placed on elderly, high-comorbidity populations, such as the ones presently treated in most European centers. Prescription of dialysis includes reaching decisions on the following elements: dialysis modality (hemodialysis (HD) or hemodiafiltration (HDF)); type of membrane (permeability, surface); and the frequency and duration of sessions. Blood and dialysate flow, anticoagulation, and reinfusion (in HDF) are also briefly discussed. The approach described in this concept paper was developed considering the following items: nutritional markers and integrated scores (albumin, pre-albumin, cholesterol; body size, Body Mass Index (BMI), Malnutrition Inflammation Score (MIS), and Subjective Global Assessment (SGA)); life expectancy (age, comorbidity (Charlson Index), and dialysis vintage); kinetic goals (Kt/V, normalized protein catabolic rate (n-PCR), calcium phosphate, parathyroid hormone (PTH), beta-2 microglobulin); technical aspects including vascular access (fistula versus catheter, degree of functionality); residual kidney function and weight gain; and dialysis tolerance (intradialytic hypotension, post-dialysis fatigue, and subjective evaluation of the effect of dialysis on quality of life). In the era of personalized medicine, we hope the approach described in this concept paper, which requires validation but has the merit of providing innovation, may be a first step towards raising attention on this issue and will be of help in guiding dialysis choices that exploit the extraordinary potential of the present dialysis “menu”.

## 1. Diet and Hemodialysis Prescription: A Necessary Integration

No treatment or schedule of dialysis is univocally recognized as superior, and, partly as a consequence, there continues to be no simple method of prescription [1]. 

Oversimplifying, we could say that dialysis is a treatment employed to remove metabolic waste, via an intra- or extracorporeal process, from the blood of people whose native kidneys are not able to perform this function [1,2,3,4]. 

Metabolic waste may be of endogenous (metabolic) or exogenous (food intake) origin, but it is food intake that ultimately regulates the patient’s anabolic and catabolic balance and also indirectly determines the level of uremic toxins, from the “simplest ones” such as urea or phosphate, to the complex, and less known “middle-molecules”, such as beta-2 microglobulin, and to some extent, parathyroid hormone (PTH) [5,6,7,8]. While there is no clear definition of the term “uremic toxins”, we will use it to identify substances present in the sera of uremic patients, with a specific detrimental effect in these subjects. According to a definition from a review by Glassock “A connection between the toxic substance and one or more of the patho-biological or clinical features of the uremic syndrome must be firmly demonstrated”. In this broad definition, therefore, “physiological” molecules” such as phosphate or potassium are also included [9].

Adequate dialysis can therefore be seen as the procedure that makes it possible to attain a stable, acceptable long-term metabolic balance in patients without sufficient kidney function. Nutritional status, which is one of the most important survival markers in dialysis patients, is a key element in the global metabolic balance [10,11,12]. Attention to nutritional markers has progressive shifted from the reduction of potentially dangerous toxins (including potassium and phosphate), to an increase in vital nutrients and prevention of long-term problems [13,14,15,16,17,18] (Table 1).

Tailoring hemodialysis requires consideration of several, sometimes conflicting aspects. Depuration has to be efficient, but highly efficient depuration may remove useful nutrients. Dialysis has to be long enough to allow for depuration of compartmentalized substances and middle molecules, but dialysis tolerance is better with shorter sessions, or with “softer” treatments. Poor dialysis tolerance is associated with poor prognosis and quality of life, particularly in elderly patients. The idea of tailored dialysis may thus be resumed in focusing on highly efficient depuration in younger patients with good nutritional status, and on high tolerance in elderly patients, for whom life expectancy is short enough not to tailor dialysis based on avoidance of long-term problems.

In a context in which no available evidence clearly supports the choice of dialysis mode (hemodialysis versus hemodiafiltration, pre- versus post-dilution, longer versus shorter durations, incremental versus standardized dialysis), dialysis prescription still relies more on personal experiences and shared views [1,2,3,4]. 

Concept papers are papers which outline personal views and personal indications, based on a subjective reading of contrasting evidence, eventually guiding treatment strategies. Along this line, this paper resumes and offers to discussion an approach to dialysis prescription based upon integration between dialysis efficiency, markers of nutritional status and comorbidity. This approach is non-validated; this limitation is however an invitation to participate to discussion on the controversial issues of nutrition markers and dialysis policy.

## 2. Tolerance beyond Depuration

The dialysis population has deeply changed in the last decades, with an impressive increase in the number of elderly patients and patients with high numbers of comorbidities. Even if the overall number of young patients has not universally decreased kidney transplantation, due to great advances in the field, is now the best mode of renal replacement therapy in this population. 

As a result, the approach to dialysis prescription, which tends towards personalization in several settings, needs to be adapted to various categories of patients that are distinct in terms of comorbidity and life expectancy. 

Attaining efficient depuration, controlled by the classic kinetic markers, may not be sufficient in all patients, and may not be the priority in the present elderly and high-comorbidity dialysis population [19,20,21,22,23]. Prescription of all treatments, including dialysis, should first of all comply with the imperative “do no harm” [24,25,26]. The high mortality on initiation of dialysis, recently termed “dialysis shock” and the lack of advantages (and possible disadvantages) of early versus late dialysis initiation, together with the contrasting results of dialysis or supportive treatment in the elderly, indicate that dialysis saves lives, but not without cost [27,28,29,30,31,32,33]. 

In this regard, the “efficiency goal”, stating that kinetic adequacy is the essential requisite for “good dialysis”, is pertinent to younger patients, but is progressively replaced by a “tolerance goal” in the elderly, where “good dialysis” should have minimal side effects [34,35,36].

This paradigm shift has also led to renewed interest in “late” and, whenever possible, incremental dialysis initiation, in which, as occurs in peritoneal dialysis, hemodialysis is prescribed with a progressive increase in the number and duration of sessions to try to minimize “dialysis shock” and preserve residual renal function [37,38,39,40,41,42]. 

Thus, “intent-to-defer” has replaced “the earlier the better” policy of dialysis initiation, and the consideration of “too much of a good thing” has convinced many physicians that dialysis efficiency should be mitigated, at least in fragile populations [20,43]. Once more, nutritional issues are central to this discussion, since malnutrition is the main marker of frailty in dialysis patients [9,10,11,12,44,45,46,47].

At the same time, we have become aware that in elderly patients a policy merely aimed at highly efficient dialysis may not always be clinically sound, and, as stated recently in another concept paper on seminars in dialysis, “less may be better”, privileging tolerance and tailored approaches in fragile patients [20].

Conversely, in the new millennium interest has increased in more frequent, high-efficiency dialysis; this option may be particularly advantageous for young patients with high metabolic demands (for example in the pregnant dialysis patient). The indications are however not sharp, and short, daily dialysis has been recognized also as a promising option for fragile patients [48,49,50,51,52].

In this changing panorama, the balance of hemodialysis prescription is shifting from standardization to personalization. Not surprisingly, different policies are followed in different settings, and similar treatments may be prescribed for different goals [53].

In an attempt to balance comprehensiveness and feasibility, we have tried to describe an approach to personalized dialysis, relying on some simple, readily available measures and markers (Table 1 and Table 2). The algorithm shown in this paper, which is the outcome of extensive discussions at two in-hospital centers (one in Italy, the other in France), was specially conceived to meet the needs of fragile and elderly patients [53]. 

**Figure 1:**
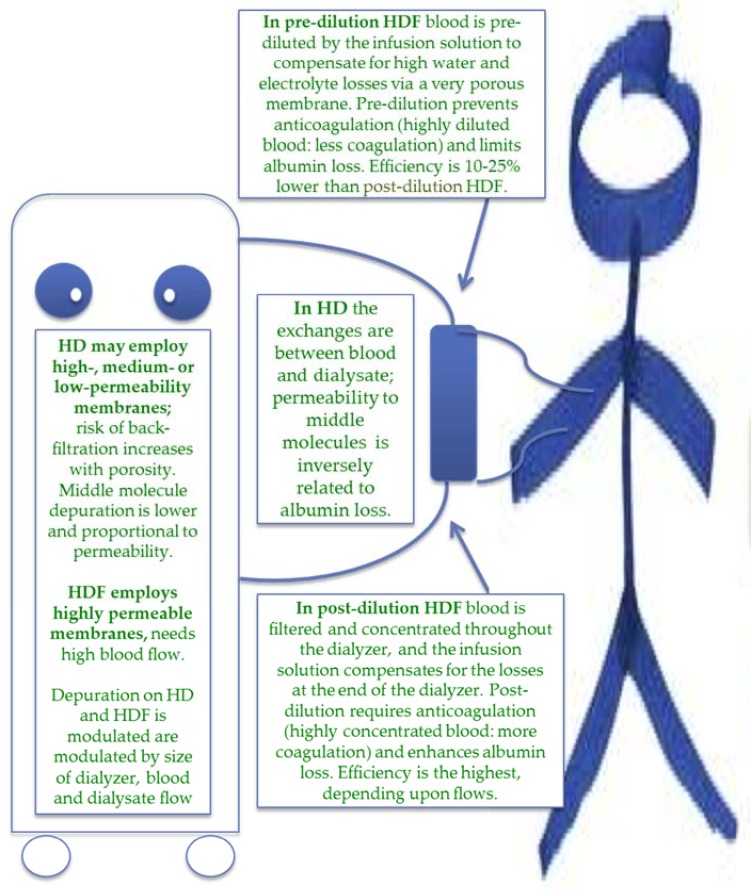
The main characteristics of hemodialysis (HD) and hemodiafiltration (HDF).

The schema is based on two options: HD (low flux) and HDF (pre- and post-dilution) (Figure 1).

The following elements were considered and integrated:

- Nutritional markers and integrated scores (albumin, pre-albumin, cholesterol; body size, Body mass index (BMI), Malnutrition Inflammation Score (MIS), Subjective Global Assessment (SGA)) [43,44,54,55,56,57,58,59,60,61,62];
- Life expectancy: age, comorbidity (Charlson Index), and dialysis vintage [60,63];
- Kinetic goals (Kt/V; n-PCR; calcium phosphate and PTH control; beta-2 microglobulin levels) [16,19,20,21,22];
- Technical aspects: vascular access (fistula versus catheter, degree of functionality, problems found) [64,65];
- Residual kidney function, weight gain [66,67,68];
- Dialysis tolerance (intradialysis hypotension, post-dialysis fatigue and subjective evaluation of the effect of dialysis on quality of life) [69].

The discussion on the prescription of dialysis includes:

- Dialysis frequency;
- Dialysis duration;
- Dialysis modality (hemodialysis (HD) or hemodiafiltration (HDF))
- Type of membrane (permeability; surface);
- Blood and dialysate flow (in HDF: pre- or post-dilutional modality);
- anticoagulation.

All these items will be discussed in a general, clinical manner, following clinical logic and guidance rather than kinetic modeling (Table 2).

## 3. Arbitrary (or Unproven) Assumptions

The algorithm proposed is based on some arbitrary, or unproven assumptions:

- Albumin is the most relevant prognostic marker in both HDF and HD [70,71,72,73];
- Albumin loss is non-selective, and low serum albumin levels have to be avoided [74,75,76,77,78] (i.e., “toxic albumin”, is not selectively lost; toxic albumin is albumin-linked to uremic toxins, for which loss should be promoted according to some authors [73,74];
- Different dialyzers in the same category are equivalent (high-, medium- or low-flux) in terms of performance and albumin leakage (while this is not entirely true, a detailed discussion is beyond the scope of this review);
- Loss of albumin is higher in the first minutes of HDF, supporting the choice of low-permeability membranes in the case of more frequent dialysis [74];
- Loss of albumin is also a marker of loss of other potentially useful nutrients, including vitamins; such a loss may contribute to malnutrition;
- Adsorption by dialysis membranes is not a relevant element in the removal of uremic toxins; if present, it is similar in similar categories of dialyzers [78].

Conversely, in this discussion we have not discussed pre-analytical and analytical errors, which may introduce further variability in the elements on which a decision is based. Furthermore, we have not considered the differences in the cost of treatments: in the past HD was often chosen in settings where the cost of HDF was significantly higher, but since the differences are leveling off in many European countries, we have considered the two treatments as equivalent.

## 4. Nutritional Markers and Integrated Scores

**Figure 2:**
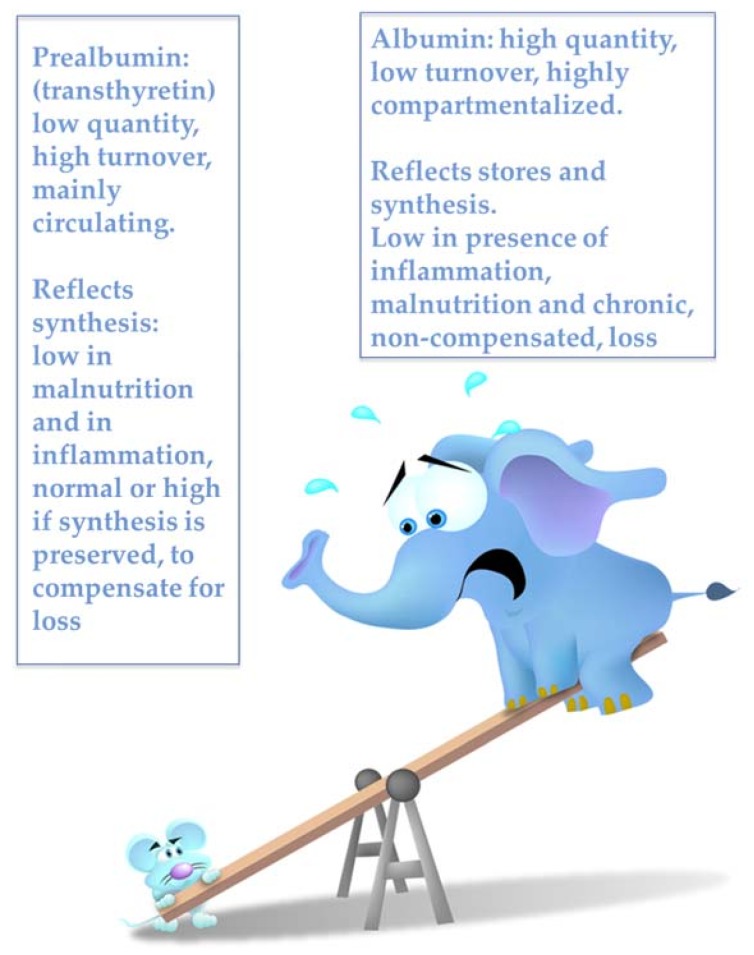
The albumin-prealbumin issue.

There are many validated nutritional markers, all characterized by serving also as mortality markers. We chose the following ones because of their simplicity, ready availability, and relatively low cost: albumin, cholesterol; body size, BMI, Malnutrition Inflammation Score (MIS), and Subjective Global Assessment (SGA) [44,45,46,61,62]. The only exception is pre-albumin, which we consider in our discussion, in spite of the fact that it has not yet been systematically integrated into the routine testing of dialysis patients (Figure 2) [79]. 

SGA and MIS are somehow complementary: the first is more sensitive to changes in nutritional status, while the latter combines nutritional and inflammation markers, giving us a potential tool to discriminate between malnutrition induced by an insufficient diet or by inadequate dialysis, which is potentially modifiable, and malnutrition resulting from inflammation and atherosclerosis, which is less likely to respond to dialysis intensification or nutritional optimization [80,81,82,83,84,85]. 

## 5. Patient Categorization

Comorbidity and nutritional status describe different categories of patients: “good” (all nutritional markers are concordant and well preserved; comorbidity is low); “bad” (all nutritional markers are altered; comorbidity is high); and “discrepant” (some nutritional markers are normal, while others are impaired, and the comorbid burden is variable). 

As previously mentioned, we considered albumin level as the leading nutritional marker, given its close relationship with survival, its availability, and its low cost [86,87,88,89,90,91]. 

The assessment of the nutritional status is further combined with comorbidity, type of vascular access and treatment tolerance, to be summarized in decisional algorithms, as will be further discussed.

## 6. Good Nutritional Status, Good Clinical Condition, Low MIS 

**Figure 3:**
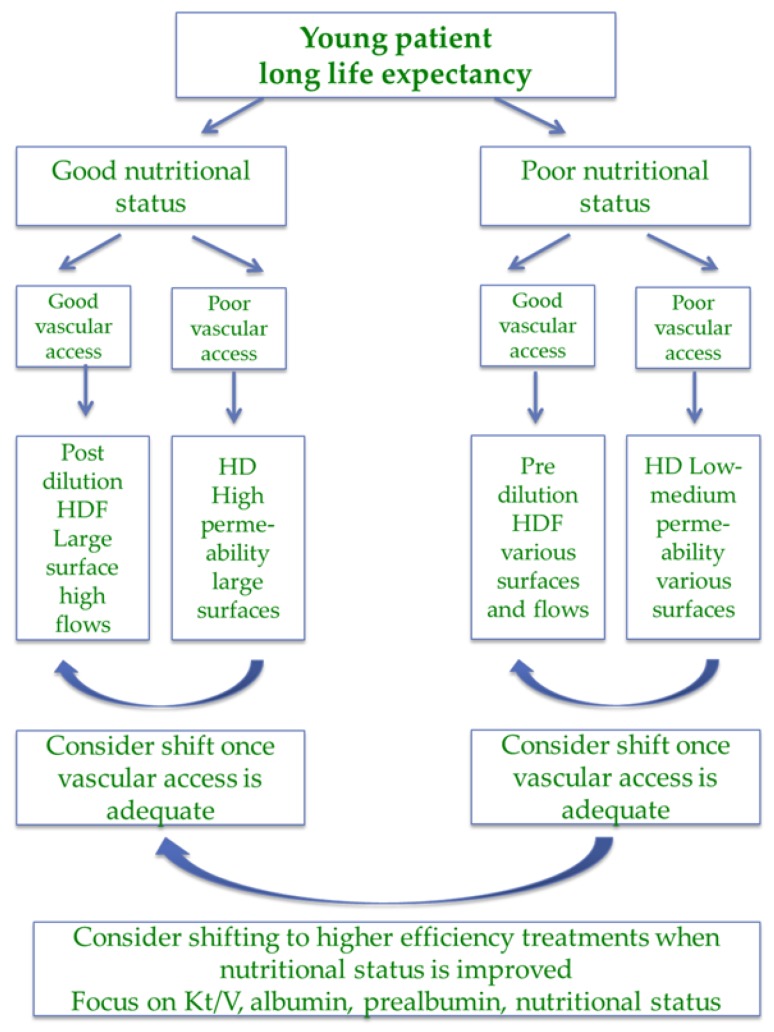
Choice of dialysis (hemodialysis (HD) or hemodiafiltration (HDF)) in the “ideal patient”.

The presence of good nutritional status, good general condition, low comorbidity, and low malnutrition inflammation score is the portrait of “the ideal dialysis patient”, a portrait less and less present in our dialysis wards due to the ageing of the population and to the selection of the fittest patients for transplantation, and even more so where the choice of preemptive transplantation is available (Figure 3).

Since this profile is associated with the longest survival, dialysis prescription should be aimed at attaining high efficiency targets. In younger patients in particular HDF may be preferable, provided ultrapure water is available given its protective effect against the risk of developing dialysis-related beta-2 amyloidosis, which is not only a severe disease, but may also be the marker of the negative effects of long-term exposure to uremia [92,93,94,95,96,97,98]. 

While the different membranes have different albumin losses, the entity of leakage, adhesion and overall loss of albumin is only partially acknowledged; albumin may indeed be seen as a marker of loss of potentially important nutrients and will be discussed as such in this paper. 

In well-nourished patients, albumin losses through high-permeability dialysis membranes are likely to be compensated by adequate production, and high-efficiency dialysis allows them to follow a less restricted diet with higher protein intake [98,99]. While some groups hold that high-permeability dialyzers allow us to obtain similar efficiency in HD and HDF, based on the current evidence, we believe that HDF is preferable, at least where cost is only marginally higher. This is particularly so in cases where significant weight loss is not an issue, as HDF prevents back-filtration, which may offset the advantages of high depuration by eliciting inflammation [100,101]. 

Since dialysis-related amyloidosis is the prototype of long-term treatment-related comorbidity, low beta-2 microglobulin levels represent a good long-term treatment marker. Japanese and French studies have demonstrated the superiority of HDF in retarding the clinical onset of amyloidosis. We consider that an investment in high-flux HDF is also worthwhile when a patient is likely to receive a transplant, even without a long wait, given the long life expectancy and considering the non-reversibility of amyloid changes after transplantation [92,93,94,95,96,97,98]. 

Policies may differ worldwide. For example, post-dilution, high-flow HDF with high-surface, high-permeability dialyzers is the main type of HDF employed in France, while the Japanese approach is milder and mainly employs pre-dilution HDF [14,20,21]. 

Following a nutrition-based approach, it is conceivable that the more aggressive strategy should be reserved to patients with good nutritional status; a compromise between removal of middle molecules and control of albumin loss may lead to pre-dilution schedules. 

The advantages of high-efficiency HDF are less evident in older patients with shorter life expectancy, in which HD may be a reasonable choice, in particular when residual renal function is present; these patients may fail compensating for albumin loss, and shorter life expectancy makes it unlikely that they will develop dialysis-related amyloidosis, which generally takes at least ten years to become clinically evident [100,101,102,103,104,105]. 

Steps to reduce albumin loss, at all ages, may also need to be taken in case of acute inflammatory events [104,105,106,107,108]. 

## 7. Poor Nutritional Status, Poor Clinical Condition, High MIS

**Figure 4:**
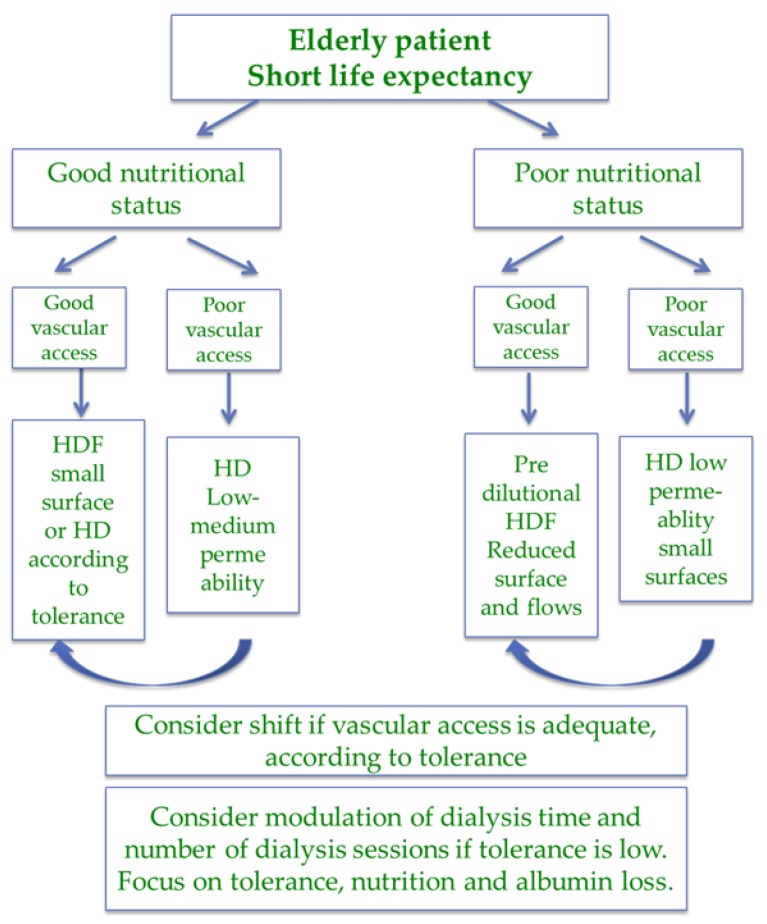
Choice of dialysis (hemodialysis (HD) or hemodiafiltration (HDF)) in the elderly fragile patient.

**Figure 5:**
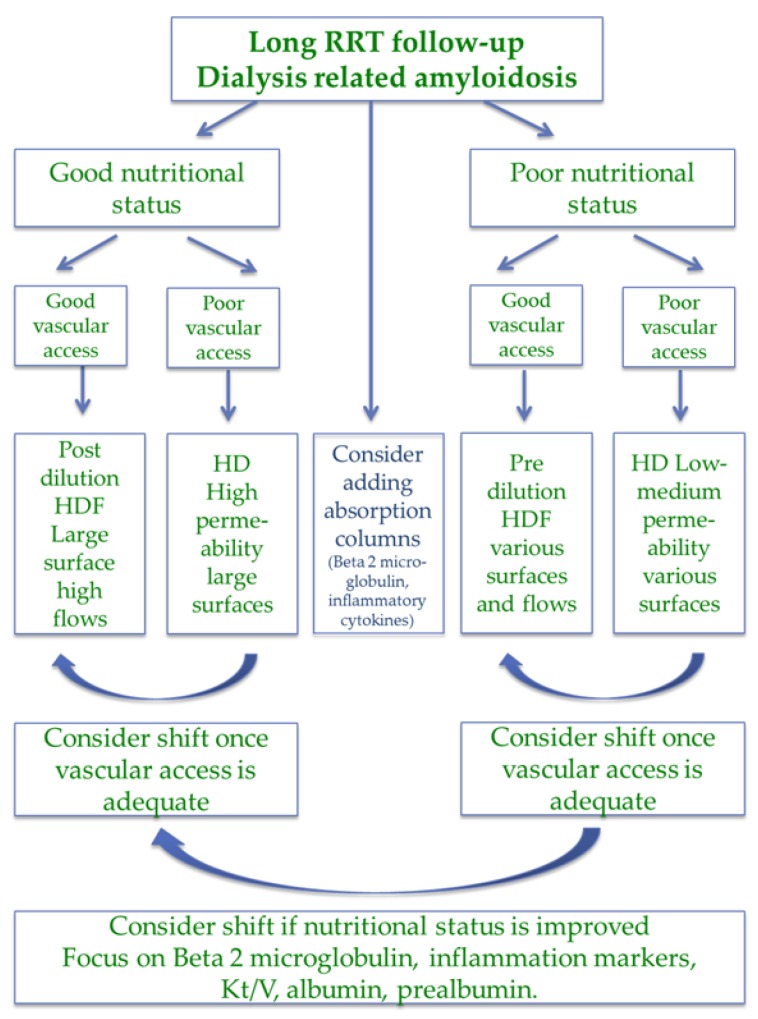
Choice of dialysis (hemodialysis (HD) or hemodiafiltration (HDF)) in the patients with long-term follow-up on renal replacement therapy (RRT).

In countries where transplantation is highly developed, the clinical profile of patients on dialysis, in particular in the hospital, is much less favorable, and the combination of old age, high comorbidity and poor nutritional status is, in general, the rule. Patients in this subset belong to three main categories: individuals with a short life expectancy and with impaired nutritional status, patients with long dialysis follow-up, and patients with acute, potentially reversible diseases, including under-dialysis or “unhealthy” dialysis initiation [41,42,43,44,109,110,111] (Figure 4 and Figure 5). 

In the first group of frail patients, with limited potential for reversing comorbidity, treatment should probably be targeted to optimal tolerance, with attention to nutrient losses [104,105,106,107,108]. 

Several policies may be pursued for high-tolerance dialysis. On a thrice-weekly schedule, HD may be less well tolerated than HDF; however, HD with low-permeability membranes has the advantage of limiting albumin and nutrient loss, and is the method of choice in short, daily dialysis, a treatment with superior tolerance [109,110,111,112,113,114,115,116,117,118,119]. 

Pre-dilution or mixed dilution HDF (with the potential advantage of reducing or avoiding the need for anticoagulants), low-volume HDF and eventually low-flux dialysis (dialysate 300–500 mL, blood 200–250 mL/min), as performed in different combination in long nightly dialysis or in short daily dialysis, should also be considered as ways to combine tolerance and limitation of nutrient loss [50,51,52,120,121,122]. Dialyzers with comparatively smaller surfaces (less than the body surface) are preferred in malnourished patients who do not have the need for high convective volumes.

**Figure 6:**
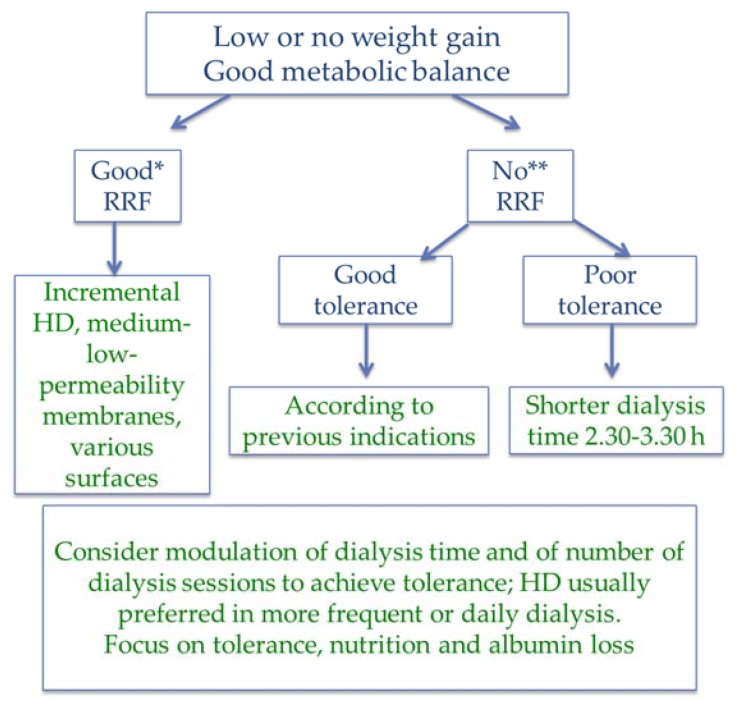
Short and incremental dialysis: “soft is best”. Good RRF (*): urine output arbitrarily defined as at least 750 mL/day, according to body surface. Survival advantage is reported for residual diuresis >250 mL/day [130,131,132]. No significant RRF (**): arbitrarily defined as urine output less than 500 mL. RRF measure: creatinine clearance or average urea and creatinine clearance (clearance 6–10 mL/min: 1 session; 3–6 mL/min: 2 sessions, modulated upon weight gain, BUN, Ca-Ph-PTH, acidosis, nutritional status, tolerance, life expectancy).

At dialysis start, incremental policies allow us to test for tolerance, starting with once- or twice-weekly sessions of 2–3 h, and progressively increasing frequency or duration according to need for increasing depuration or ultrafiltration and to tolerance [37,38,39,40,41,123,124,125,126,127,128,129] (Figure 6). 

Since tolerance is strictly linked to dialysis duration, we consider that a reasonable policy may be that of starting with relatively short dialysis sessions (2–3 h), progressively increasing the number, up to three sessions, and deciding, when needed and based upon tolerance, if shifting to the classic 12 h per week divided into three sessions of 4 h or into four sessions of 3 h. A similar policy of small session may be proposed in patients already on dialysis but with poor tolerance, severe hypertension, or who need intensive metabolic correction (Figure 6).

The use of high-permeability membranes in HD is currently the choice in many settings, although the risk of back-filtration is far from negligible, in particular when weight loss is minimal. While it is true that using ultrapure water should offset the risks involved, the advantages, if any, of are not clear [133].

In patients starting dialysis with a potentially reversible impairment of nutritional status, we usually prefer to start with HD with low-permeability membranes, and to shift them to high-efficacy HDF to increase middle-molecule depuration as soon as the clinical situation has stabilized and nutritional markers (first of all prealbumin) start ameliorating.

The new generation of dialyzers with selective permeability to middle molecules may help solve this dichotomy, if they maintain the promised long-term benefits [130,134,135].

## 8. Discrepant Measures of Nutritional Status, Clinical Condition, MIS

Dialysis patients are not easily described by a black-and-white, contrasted picture, and intermediate pictures are often present; the most common one, at least in our settings, is the combination of obesity, high comorbidity and high MIS index, with or without low albumin levels.

Mortality and obesity are linked in a paradoxical relationship [131,132,136,137,138,139,140,141,142,143,144,145]. A high BMI is not necessarily a sign of good nutrition, and obese sarcopenia may mask significant protein malnutrition [139,140,144]. Impedance analysis can help us identify sarcopenic obesity, but data have to be considered with caution, since impedance is less precise in obese patients [140]. 

It may be difficult for patients with a high BMI to reach the efficiency target, even if adjusted Kt/V is followed [143,144]; in cases in which the attainment of metabolic targets is difficult, more frequent dialysis should be considered, even if formal demonstration of an advantage is lacking. 

Among dialysis patients, those with a low BMI usually have a poor nutritional status, even if some exceptions may occur patients who are active or practice a sport. Conversely, an extremely low BMI is invariably a sign of poor nutrition, and a low BMI is therefore considered to be one of the general indexes of malnutrition [146,147,148].

Albumin is a “leading biomarker”, integrated into prognostic scores, including MIS and the dialysis-adapted Charlson Index [146,147,148,149]. Discrepant patterns are sometimes found, however. The most common one observed in our practice is the association between low albumin levels, normal or high BMI, normal cholesterol, and normal pre-albumin levels [53]. 

Pre-albumin (or transthyretin) is a reliable short-term marker of protein synthesis, sensitive, as albumin is, to inflammation, acute diseases and critically reduced food intake [146]. Normal pre-albumin associated with low albumin levels suggests an anabolic phase or chronic albumin losses (including intradialytic loss and ascites). Several other makers of protein turnover have been proposed, including complement proteins, immunoglobulin levels, uric acid, or more recently Klotho, fibroblast growth factor 23 (FGF23), and osteoprotegerin (OPG), but their use has not yet been codified in the clinical practice [150,151,152,153,154,155,156,157]. 

## 9. Vascular Access and Anticoagulation

Well-functioning vascular access is of obvious importance in dialysis and is a requisite for HDF [158,159,160,161,162,163]. While the imperative “fistula first” is still valid for patients with a longer life expectancy, poor vascular patrimonies, severe cardiac disease, chronic hypotension, hypercoagulability, and the failure of previous artero-venous (AV) fistulae or grafts increase the need for permanent catheters. In older patients, a distal, native AV fistula may not be feasible. Algorithms based on age and comorbidity now often privilege permanent catheters in elderly patients with a short life expectancy and suggest considering prosthetic or proximal fistula as a first option in patients with intermediate characteristics [164,165,166,167,168]. 

Even if malfunction is not a prerogative of indwelling catheters, low flows are common, in particular if these catheters are the rescue choice in patients with previous failures of an AV fistula or graft. In this context, HD may be a more reasonable choice, in the presence of lower blood flow (Figure 2, Figure 3 and Figure 4).

The need for anticoagulation is an important issue: besides cases with anti-heparin antibodies or hemorrhagic disorders, in which heparin has to be avoided, avoiding or minimizing heparin reduces hemorrhagic risks, in particular for elderly patients treated with antiplatelet agents and anticoagulants [169,170,171]. Fistula malfunction increases the risk of intradialytic coagulation, in particular in post-dilution HDF. Predilution HDF is a very good choice in patients with a well-functioning AV fistula, while HD is more manageable at low blood flows, but heparin administration may need to be increased; shorter and more frequent HD can be performed without anticoagulation. Heparin-coated membranes should be considered in selected cases [172,173].

## 10. Dialysis Initiation and Residual Renal Function

The preservation of residual renal function correlates positively with survival. In this context, incremental dialysis combines the advantages of better preservation of the kidney function with lower dialysis-related morbidity, and lower costs [174,175,176,177]. 

The high mortality seen during the initial period of dialysis and the lack of advantages associated with “early” dialysis initiation suggest that a cautious increase in treatment time and frequency could be advantageous; the approach is not agreed and one large retrospective study suggests that dialysis in facilities that practice a more aggressive dialysis start (first session) is associated with better survival [178] (Figure 6).

While HDF has been reported to better preserve residual renal function, the evidence is scant, and the most common approaches are based on short HD, progressively increasing in duration and/or frequency [37,38,39,40,41,42]. In fact, it has been found that post-dialysis fatigue and intradilalytic tolerance correlate with the duration of sessions [179]. 

The lack of agreed approaches and kinetic targets and the fact that there is no agreed way to assess residual clearances, make it difficult to assess the adequacy of incremental dialysis. Bearing this in mind, in prescribing an increase in dialysis frequency or duration in the context of incremental dialysis, we follow the same clinical criteria that induce us to start dialysis treatment, namely hypertension, weight gain, anemia, nutritional status, and calcium phosphate, PTH and acid-base balances. 

Decisions may be difficult in patients with nephrotic syndrome, in which the advantages of offsetting proteinuria by vigorous dehydration, combined with Angiotensin converting enzyme inhibitors and/or angiotensin receptor blockers when necessary, have to be balanced against loss of renal function; no study, to the best of our knowledge, has specifically addressed this issue, and decisions should be made case per case.

Patients with failing kidney grafts also pose further challenging problems: signs of protein wasting are frequent, in particular in patients on long-term steroids; criteria for dialysis initiation are otherwise not different before transplantation and after graft failure [180,181,182]. The question of when and how to discontinue antirejection drugs still needs to be clarified: while the trend was to rapidly discontinue antirejection drugs as soon as dialysis was started, the trend is now to keep them at low doses at least in cases in which a further graft is possible. In these often fragile patients, increased susceptibility to infections and chronic inflammation (and possibly also chronic rejection) can counteract efforts to improve nutritional status, even in the presence of high dialysis efficiency. 

**Figure 7:**
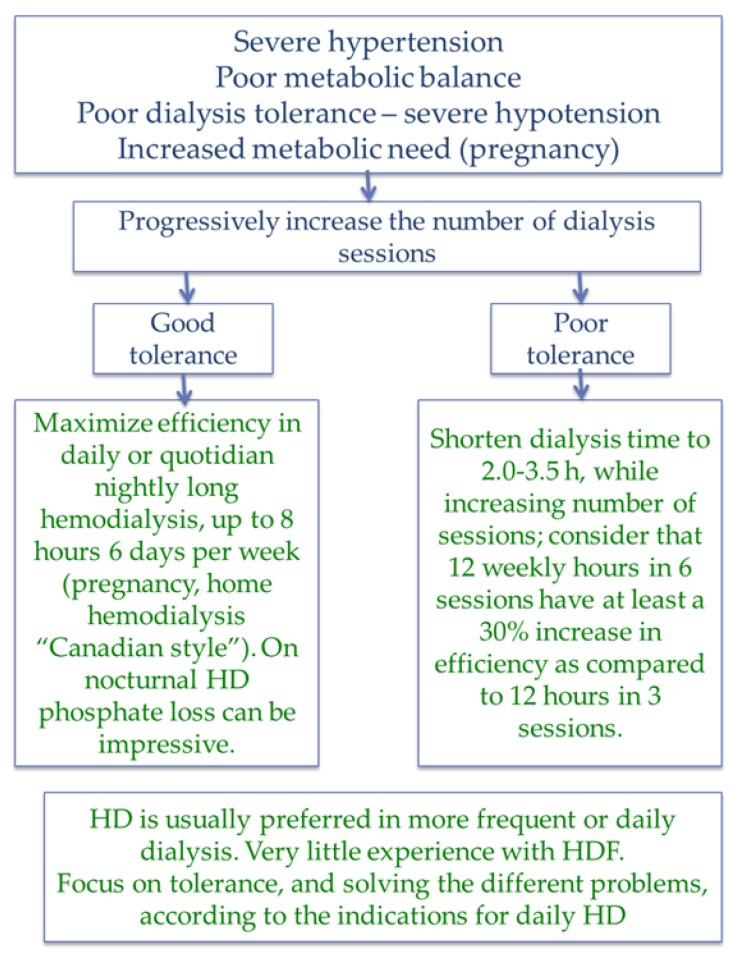
Daily and more efficient dialysis.

Conversely, daily dialysis and highly efficient dialysis may offer interesting solutions to two opposite problems: low tolerance, overcome by shortening the sessions, and need for higher efficiency, by increasing time and frequency (Figure 7).

## 11. What This Review Did Not Address

This review was undertaken to serve as a basis for discussion on dialysis prescription, in particular for those cases escaping the classic “efficiency rules”, namely elderly patients with high comorbidity. The decisional pathway was established employing common markers, most of them controlled in our monthly “dialysis profiles”. 

In our search for a simple approach, we did not consider many important points, including in-depth diagnosis of protein energy wasting, the psychological aspects of malnutrition and its link with depression. Furthermore, we did not include certain important elements in the decisional strategy, such as potassium and bicarbonate, fluid and sodium, blood pressure and brain natriuretic peptide (BNP). We have not dealt with nutritional interventions in dialysis patients, a complex issue beyond the scope of the present review, which focuses on dialysis prescription within the context of a policy of liberal food intake, considering that losses in dialysis should be compensated for by a rich and varied diet, while restrictions may lead to malnutrition instead of improving metabolic parameters.

We are well aware of the limits of our review, which describes an approach to dialysis prescription and the criteria it is based on.

As all concept papers, this working hypothesis awaits validation. We await a longitudinal analysis but until such time as one is undertaken, we would like to have feedback from readers on what they see as the strong and weak points of our hypothesis.

## 12. Conclusions and Suggestions for Future Research

The present concept paper offers a personal interpretation of the current evidence from the pragmatic point of view of dialysis prescription, modeled upon life expectancy and nutritional status.

A longitudinal study is needed to validate this pragmatic approach and to highlight its limits and advantages, in particular in elderly and high-comorbidity dialysis populations.

## Figures and Tables

**Table 1:** “Magic numbers” employed in nutritional evaluation and dialysis prescriptions and their limits: some laboratory tests.

Item	Magic numbers	Pros	Cons
Albumin	Normal ≥35 g/L to ≥40 g/L, may differ according to European or U.S. standards.	Simple, readily available, low cost, validated.	Depends on hydration, sensitivity to losses (especially in HDF or HD with high-permeability membranes). Validated in HD with thrice-weekly schedules.
Pre-albumin	Normal (depending on laboratory); in general 0.18–0.35 g/L.	Influences the evaluation of albumin levels.	Relatively expensive, but not fully validated, high variability. Little information for elderly patients.
Cholesterol	Usual threshold for malnutrition: <150 mg/dL.	Simple, readily available, low cost, validated.	Several metabolic interferences, not evaluable in the case of specific treatments.
Kt/V	Threshold for adequate dialysis depends on the formula chosen; adequate dialysis is usually defined as a level >1.2–1.4 in thrice-weekly dialysis.	Simple, readily available, validated, low cost.	Depends on formula, day of the week (first vs. midweek dialysis), baseline urea level; post-dialysis sample may be affected by urea rebound; may be higher in malnourished patients (low volumes). No fully validated formula for less and more frequent dialysis.
n-PCR	Threshold for adequate protein intake depends on the formula chosen; adequate intake usually >1.2 g/kg/day in thrice-weekly dialysis.	Simple, readily available, validated, low cost.	The best protein intake in elderly patients is not clear; data were established for relatively young patients when ideal intake was set at 1 g/day in the overall population (presently 0.8); does not distinguish between catabolism and intake.

Kt/V: mathematical formula relating urea level before and after dialysis. n-PCR: normalized protein catabolic rate; HDF: hemodiafiltration; HD: hemodialysis.

**Table 2:** “Magic number” definitions and limits: hemodialysis (HD) and hemodiafiltration (HDF) prescriptions.

Item	Number Definition	Advantages of the Definition	Disadvantages/Limits of Standardization
Permeability	Usually defined as high, medium, or low with respect to middle-molecule depuration; different cut-points available, no fully agreed definition.	Clear and easy definition; all types of membranes can be used in HD, and only high-permeability membranes in HDF. Back-filtration in HD is proportional to permeability.	Differences are less sharp for new membranes; research to improve selectivity, differences between membranes in the same category may be relevant.
Membrane size	In square meters: usually related to body surface (lower/higher/equal).	Clear and easy; several surfaces usually available for each membrane type.	Membrane size is related to membrane type and anticoagulation; effect of size on depuration depends on membrane performance.
Blood flow	No fully agreed standard; European reference 300–350 mL/min; in other settings target flow may be as high as 450 mL/min.	Clear and easy definition; good blood flow is also a marker of correct functioning of the vascular access.	Target may vary according to vascular access and type of treatment (lower in long-hour dialysis). Highly dependent on vascular access.
Dialysate flow	No fully agreed standard; European reference 500 mL/min., may be as high as 800 mL/min.	Clear and easy definition; agreed international standard.	Prescription can be adjusted (higher in HDF, lower in some types of daily dialysis).
Reinfusion (HDF)	No fully agreed standard; European reference 24 L/session on HDF.	Clear relationship between exchanges and middle-molecule depuration.	Standards are different across the world; pre-/post-dilution protocols are different; loss of albumin may increase with high exchanges.
Number of dialysis sessions	Thrice-weekly; incremental: 1–2 per week with progressive increase; intensive: 4–7 per week. “daily dialysis” at least 5 per week.	Clear, simple, validated.	All frequencies that differ from thrice-weekly are less validated, protocols are highly center-dependent.
Dialysis duration	Standard: 4 h thrice-weekly; shorter in “short” daily dialysis; various combinations of 2–8 h and 1–7 sessions.	Clear, simple, validated.	All durations that differ from 4 h are less validated, protocols are highly center-dependent.
